# Species Diversity and Distributional Pattern of Cockroaches in Lahore, Pakistan

**Published:** 2017-05-27

**Authors:** Hafsa Memona, Farkhanda Manzoor, Saffora Riaz

**Affiliations:** Department of Zoology, Lahore College for Women University, Lahore, Pakistan

**Keywords:** Diversity, Cockroach, Pakistan, *P. americana*, *B. germanica*

## Abstract

**Background:**

Cockroaches are found as the most common urban pests of tropical countries, prompting economic and serious health risk problem for humans by carrying microbes and allergens, acting as vector for various pathogens of diseases. The present study was conducted from April 2013 to March 2014 in various human dwelling localities of urban area of district Lahore, Pakistan.

**Methods:**

Cockroaches were collected randomly by hand, food baited and sticky traps throughout the year. Four species of cockroaches (*Periplaneta Americana* (*P. amercana*), *Blattella germanica* (*B. germanica*), *Blatta orientalis* (*B. orientalis*), and *Blatta lateralis* (*B. lateralis*) were collected and identified from the study site*.*

**Results:**

*B. germanica* was the most dominant indoor species with highest diversity indices in study areas. Overall cockroach species diversity was highest in July–September, 2013 with highest Simpson index of diversity and Shannon index as well. *P. americana* was found second broadly distributed in the study area followed by *B. orientalis* and *B. lateralis* were intermediately distributed in residential areas and narrowly distributed in hospitals. Residential areas and hospitals were highly infested with *B. germanica* followed by *P. americana*. Population index of *B. germanica* for hospitals was double than residential areas. *B. lateralis* was observed as displacing *B. orientalis* in outdoor habitat through competing with its habitat and food sources.

**Conclusion:**

The infestation rate of different species depends on availability of food sources, sanitary conditions and climatic conditions. Cockroach infestation can be controlled with knowledge about their biology and behavior, attention to sanitation and effective use of commercial insecticides.

## Introduction

Out of the 4600 species of cockroaches only 50 species have been reported as pests of human structures and dwellings worldwide. They randomly infringe in human habitation and never cause threat to indoor structures (Cochran et al. 1980, Bonnefoy et al. 2008). Morphologically cockroaches are characterized by their dorsoventrally flattened body, head concealed beneath the pronotum, chewing mouth parts, prominent antennae and cerci. They are placed recently in their own order of Blattodea (Triplehorn and Johnson 2005).

All types of human habitations including hospitals and houses are significantly infested with cockroaches. Highly populated houses and impoverished living settings are breeding sites for indoor species especially *Blattella germanica* (*B*. *germanica*) (Bonnefoy e al. 2008). Poor sanitation, disrepair and clutter contribute to increase population of cockroaches in human habitat. Foodstuffs can be contaminated with cockroach feces, body parts and pathogens. Increased population of cockroaches inside the homes will cause asthmatic allergies in peoples which can be life threatening in some cases (Lamiaa et al. 2007).

The predominant species of cockroaches found in various types of human dwellings in Bangkok, Thailand, Kuala Lumpur Federal Territory, peninsular Malaysia, Singapore, China, Indonesia, India and Pakistan are: Family Blattidae include *Periplaneta americana* (*P. americana*) (American cockroach), *P. australasiae* (Australian cockroach), *Periplaneta brunea (P. brunnea)* (Burmeister) (Large brown cockroach), *Periplaneta fuliginosa* (*P. fuliginosa)*, *Blatta orientalis* (*B*. *orientalis*) (Oriental cokroach), *Neostylopyga rhombifolia* (*N*. *rhombifolia*) (Stool) The Harlequin cockroach, *Blatta* (*Shelfordella*) *lateralis* (*B*. *lateralis*) (Walker) (Turkistan cockroach) and *Hebardina concinna* (*H*. *concinna*) (Dehaan) (Sriwichai et al. 2002, Chompoosri et al. 2004).

Family Blattelidae include *B. germanica* (German cockroach), *Blattella lituricollis* (*B. lituricollis*) (Walker) (Smaller German cockroach), *Supella longipalpa* (*S*. *longipalpa*) (Fabricius) (Brown banded cockroach), *Blattella vaga* (*B. vaga*) Hebard (Field cockroach), *Blattella asahinai* (*B. asahinai*) Mizukubo (Asian cockroach), *Jacobsonina erebis* (*J*. *erebis*), *Symploce pallens* (*S*. *pallens*) (Smooth cockroach) *Symploce sphaerica* (*S. sphaerica*), *Symploce miyakoensis* (*S. miyakoensis*), *Symploce okinerabuensis* (*S. okinoerabuensis*), *Symploce paramarginata* (*S. paramarginata*) and *Symploce evidens* (*S. evidens*). Family polyphagiadae include *Polyphaga aegyptica* (*P*. *aegyptica*) and *Polyphaga saussurei* (*P. saussurei*). Family Blaberidae include *Rhyparobia maderae* (*R*. *maderae*) (Madeira cockroach). Family Pycnoscelidae include *Pycnoscelis surinamensis* (Linnaeus) (*P*. *surinamensis*) the Surinam cockroach. Family Oxyhaloidae include *Nauphoeta cinerea* (Olivier) (*N*. *cinerea*) the lobster cockroach (Cochran et al. 1980, Zahedi and Jeffery 1996, Boyer and Rivault 2004, Jeffery et al. 2012, Wang and Che 2013).

Different studies in Pakistan confirms the presence of *P. americana*, *B. orientalis* and *B. germanica* species of cockroaches in Pakistan (Mlso et al. 2005, Saira 2005, Ahmed et al. 2010, NIH 2010, Wakil et al. 2012, Malik et al. 2013, Syed et al. 2014).

Cockroaches are one of the most important agent which can transmit almost 60 species of yeast, 150 bacterial species, 45 species of parasitic worms and 90 species of protozoa to human life either biologically or mechanically (Tachbele et al. 2006, Al-Marjani et al. 2008, Saichua et al. 2008, Al-bayati et al. 2011, Akinjogunla et al. 2012, Tilahun et al. 2012, Goralska and Kurnatowski 2013, Vazirianzadeh et al. 2014). They get infected with pathogenic bacteria causing bubonic plague, leprosy, dysentery, urinary infections, abscesses and pimples etc. bacterial species can stay alive on cockroach body surfaces for many days (Vahabi et al. 2007).

The parasitic organisms identified from cockroaches include helminthes and protozoans. The helminthes include *Strongyloides stercoralis* (*S*. *stercoralis*), *Ascaris lumbricoides* (*A*. *lumbricoides*), *Trichuris trichura* (*T*. *trichura*), *Taenia* spp and identified protozoans are *Cyclospora* spp, *Entamoeba histolytica* (*E*. *histolytica*), *Entamoeba coli* (*E*. *coli*), *Balantidium coli* (*B*. *coli*) and *Isospora belli* (*I*. *belli*) (Thyssen et al. 2004, Salehzadeh et al. 2007, Nyarango et al. 2008, Chamavit et al. 2011, El-Sherbini and El-Sherbini 2011). Cockroaches are contaminated with medically important fungi including *Candida*, *Aspergillus niger* (*A*. *niger*), *Mucor*, *Rhizopus* spp, *Aspergillus fumigans* (*A*. *fumigans*) and *Penicillium* spp. (Tatfeng et al. 2005, Salehzadeh et al. 2007).

Cockroach associated bacteria include *Klebsiella pneumoniae* (*K*. *pneumoniae*), *Salmonella* spp, *Escherichia coli* (*E*. *coli*), *Pseudomonas aeruginosa* (*P*. *aeruginosa*), *Enter-obacter cloacae* (*E*. *cloacae*), *Citrobacter freundii* (*C*. *freundii*), *Enterobacter aerogenes* (*E*. *aerogenes*) and *Proteus mirabilis* (*P*. *mirabilis*) which are potential pathogens. There was no significant difference between the overall bacteria load on the external surface in cockroaches found in the food-handling establishments (60.08%) and households (39.92%) (Wannigama et al. 2014). *Pseudomonas aeruginosa* has been demonstrated to multiply in the gut and excretion of the bacteria continued up to 114 days. Further studies also revealed that *Salmonella typhi* (*S*. *typhi*), *Shigella dysenteriae* (*S*. *dysenteriae*) and toxigenic strains of *E. coli* retained in the gut of cockroaches for up to several days (Fotedar et al. 1993, Pai et al. 2003).

Present study was conducted with the objective to report species composition, diversity, abundance, richness and population dynamics of cockroaches in urban areas of district Lahore. Lahore features a five season semi-arid climate ie foggy winters (15 Nov–15 Feb) with few western disturbances causing rains, pleasant spring (16 Feb–15 April), summer (15 April-June) with dust rain storms and heatwave periods, rainy monsoon (July–16 September) and dry but pleasant autumn (16 September–14 November) (Punjab Meteorology Department 2014). Due to the high population density in the city, 5000 tons per day of solid waste is produced in Lahore that is collected and disposed in one landfill site at Mehmood Booti and sewage is drained into river Ravi (Lahore Waste Management Company Report 2014). There are few scattered researches were conducted about species composition and abundance in last years. Economic importance of this domestic pest should be recognized. However, they are notorious as vector of nosocomial infections, contaminating and deteriorating food and consumables, carry allergens of asthma and resistant to insecticidal sprays.

## Materials and Methods

### Experimental site

Study site is the urban area of district Lahore lying between 31°15′–31°45′ N and 74°01′–74°39′ E, 217m elevated from sea level with total population of 7,566,000. Samples were collected from different hospitals, shopping malls/stores, institutes and residential areas in different seasons around one year.

### Sampling and identification of cockroaches

Cockroaches were observed and collected randomly from April 2013 to March 2014 with the help of sticky traps, food baited pitfall traps and manual catching by hand. Specimens were collected from 20 different sites including hospitals (Punjab Institute of Cardiology, Mayo Hospital, Sheikh Zaid Hospital, Jinnah Hospital and General Hospital) shopping malls/stores (Swera departmental store, Metro cash and carry and Hyperstar), institutes/office (LCWU, Punjab university, UVAS, GCU Lahore and PASSCO), houses (Mughalpura, Model town, Shadman, Shalmar, Maraghzaar, Johar town and Jallo town). Global Positioning System of study sites are listed in Table 1. Traps were kept on the floor close to the wall of room, under cupboards, bed, storage racks, under washbasins and pantry. Each trap was placed in living room, bedroom, bathroom and kitchen of houses, different wards, store rooms, kitchen stores, canteen area of hospitals, in grocery area, food area of shopping store and in each working room of the Institute/office units for three consecutive nights. Nymphs along with adults were heavily trapped in baits and traps. The collected specimens were transported to entomology laboratory for identification up to species level with the help of published keys (Pratt and Littig 1969, Abul Hab 1980, Hagenbuch et al. 1988, Roth 1995, Choate 2009). Species abundance and richness was evaluated in 4 trimesters comprising whole year from April, 2013 to March, 2014.

**Table 1.** Global Positioning System location of study sites in urban areas of Lahore**,** Pakistan

**Serial. Number**	**Study site**	**Latitude**	**Longitude**
**1**	Punjab Institute of Cardiology	31°32′18.48″N =31.5384667	74°20′9.28″E =74.3359111
**2**	Mayo Hospital	31°34′18.06″N =31.5716833	74°18′57.04″E =74.3158444
**3**	Shaikh Zayed Hospital	31°30′29.82″N =31.5082833	74°18′30.17″E =74.3083806
**4**	Jinnah Hospital	31°29′3.93″N =31.484425	74°17′48.40″E =74.2967778
**5**	General Hospital	31°27′17.46″N =31.45485	74°21′0.94″E =74.3502611
**6**	Swera departmental store	31°25′54.81″=31.4318917	74°17′11.38″=74.2864944
**7**	Metro cash & carry	31°29′34.65″N =31.4929583	74°25′1.57″E =74.4171028
**8**	Hyperstar	31°32′5.69″N =31.5349139	74°21′47.79″E =74.363275
**9**	LCWU, Lahore	31°32′41.85″N =31.5449583	74°19′37.97″E =74.3272028
**10**	Punjab University, Lahore	31°29′44.24″N =31.4956222	74°17′39.17″E =74.2942139
**11**	UVAS, Lahore	31°34′29.03″N =31.5747306	74°17′57.48″E =74.2993
**12**	GCU, Lahore	31°34′22.14″N =31.5728167	74°18′29.22″E =74.3081167
**13**	Passco office	31°33′36.68″N =31.5601889	74°19′56.62″E =74.3323944
**14**	Mughalpura	31°33′46.53″N =31.562925	74°22′49.35″E =74.380375
**15**	Model town	31°28′37.18″N =31.4769944	74°19′44.66″E =74.3290722
**16**	Shadman	31°32′14.97″N = 31.5374917	74°19′50.76″E = 74.3307667
**17**	Shalamar town	31°35′12.47″N = 31.5867972	74°22′55.29″E = 74.382025
**18**	Maraghzaar colony	31°29′44.24″N =31.553326	74°17′39.17″E =74.305122
**19**	Johar town	31°27′43.38″N = 31.46205	74°17′38.90″E = 74.2941389
**20**	Jallo town	31°35′47.57″N = 31.5965472	74°29′57.97″E = 74.4994361

### Weather Data Collection

Average monthly temperature and humidity data were obtained from the Punjab Meteorological Department, Lahore and comparatively analyzed with population density of cockroaches.

### Data analysis

The observations were tabulated and data were statistically analyzed using Microsoft excel. Relative Abundance, species richness and evenness of each species of cockroaches was calculated. Diversity of different cockroach species on the Simpson and Shannon indices was worked out according to Simpson (1949) and Shannon-Weiner function (Odum, 1975). Species relative abundance was compared with average monthly temperature and relation between population density and change in temperature was determined.

## Results

### Identified species of cockroaches

From different types of human dwelling localities 4 species of cockroaches belonging to two families (Blattidea and Blattelidae) were identified during the entire sampling period.

1. American Cockroach (*P. americana*) They found in sewers and basements especially around pipes and drains.
2. Oriental Cockroach (*B. orientalis*): They found beneath the mulch, leaf litter, stones and debris outdoors, garbage, filthy materials that is going to decay.
3. German Cockroach (*B. germanica*): They found in kitchens, storage areas especially where food being prepared or stored.
4. Turkestan Cockroach (*B. lateralis*):

Turkestan cockroaches are native to large area of the Middle East extending from Libya Eastward to Central Asia including Afghanistan, Pakistan, Uzbekistan and southern Russia (Alesho 1997). They were found in compost piles, gardens, potted plants and homes with clay floors.

### Distribution and abundance of cockroach species

From this study it was revealed that *B. germanica* was the most dominant species belonging to family Blattelidae comprising of 45% of the total catch followed by *P. americana* (35%) belonging to family Blattidae. These two species comprises 80% of the total specimens collected. Other two species include *B. orientalis* (9%) and *B. lateralis* (11%) of the total specimen trapped. These findings are compatible with the studies of Sandhu and Sohi (1981). From all the specimens captured during study period 62% (8328) specimen were at different nymphal stages, 27% (3626) adult male and adult females are 11% (1479). The number of females was lower as compared to males in traps because females are less agile and are often hidden in deep crevices, engaged in reproductive activity.

During the first trimester of study (April–June, 2013) the minimum and maximum average temperature was 23.8 °C and 37.6 °C, respectively and relative humidity was 41% (Table 3). The autumn was turning to end and summer was started. This is the most compatable season of breeding of eggs and nymphs. In this trimester, nymph collection was more prominent as compared to adult males and females. Most dominant species (46.78%) of all catches was *B. germanica*, followed by *P. americana* (33.50%) while *B. orientalis* and *B. lateralis* are 9.96% and 9.75% respectively (Table 2). Diversity indices were worked out for all species found in 20 different sites revealed Shannons index value of −0.51185 and Simpson’s index of diversity was 0.6495. The dominant species of first trimester was *B. germanica* with a value of 0.218879 (Simpson index) and −0.15434 (Shannon index). During sampling the higher number of males in food bait traps and sticky traps was not surprising as the males were active predators and roam in search of mates. Females were low in number because they conserve energy for reproduction and found less active and common. One of the many reason is may be the traps were baited with female sex attractants which results in maximum male capturing in traps.

**Table 2.** Diversity indices of different cockroach species collected from April 2013–March 2014

**Trimester**	**Species**	**No. of Cockroaches**	**Percentage %**	**Relative abundance Pi**	**Shannon index Pi (lnPi)**	**Simpson index Pi^2^XS**	**Species Evenness (S) H/logS**
**1^st^**	*P. americana*	1573	33.50	0.3349	−0.15911	0.112202	−0.51185/0.60206 = −0.85016
	*B. germanica*	2197	46.78	0.4678	−0.15434	0.218879	
	*B. orientalis*	468	9.96	0.0996	−0.09981	0.009932	
	*B. lateralis*	458	9.75	0.0975	−0.09859	0.009512	
	Total	4696			H= − 0.51185	D= 0.3505, 1-D= 0.6495	
**2^nd^**	*P. americana*	2123	35.81	0.358191	−0.15971	0.128301	−0.51389/0.60206 = −0.85355
	*B. germanica*	2629	44.36	0.443563	−0.1566	0.196748	
	*B. orientalis*	481	8.11	0.081154	−0.08851	0.006586	
	*B. lateralis*	694	11.7	0.117091	−0.10907	0.01371	
	Total	5927			H= − 0.51389	D= 0.3453, 1-D= 0.6547	
**3^rd^**	*P. americana*	671	36.03	0.360365	−0.15973	0.129863	−0.51187/0.60206 = −0.85019
	*B. germanica*	831	44.62	0.446294	−0.15637	0.199179	
	*B. orientalis*	162	8.70	0.087003	−0.09226	0.00757	
	*B. lateralis*	198	10.63	0.106337	−0.1035	0.011308	
	Total	1862			H= − 0.51187	D= 0.3479, 1-D= 0.6521	
**4^th^**	*P. americana*	351	37.02	0.370253	−0.15976	0.137087	−0.50419/0.60206 = −0.84066
	*B. germanica*	387	40.82	0.408228	−0.15884	0.16665	
	*B. orientalis*	68	7.17	0.07173	−0.08208	0.005145	
	*B. lateralis*	142	14.97	0.149789	−0.1235	0.022437	
	Total	948			H= − 0.50419	D= 0.4313, 1-D= 0.5687	

**Table 3.** Trimester average temperature minimum and maximum and relative percentage of humidity recorded from April 2013 to March 2014

**Trimester**	**Average Temperature °C**		**Average relative humidity %**	**No. of cockroaches trapped**	**Percentage %**

	**Minimum**	**Maximum**			
**1^st^ (April–June)**	23.8	37.6	41	4696	35
**2^nd^ (July–Sep)**	24.8	34.1	74	5927	44
**3^rd^ (Oct–Dec)**	13	26.5	68	1862	14
**4^th^ (Jan–March)**	9	21.4	68	948	7

During the second trimester of study (July–September, 2013) the minimum and maximum average temperature was 24.8 °C and 34.1 °C, respectively and relative humidity was 74% (Table 3). Summer season was at peak and cockroaches were mostly found in indoor sheltered areas. This is the most favorable season of metamorphosis of nymphs to change into adults. Different species have different life spans and time period to turn nymphs into adults varies among different species. In this trimester, late nymph collection was comparable to adult males and females. More dominant species (44.36%) of all catches *B. germanica* was followed by *P. americana* (35.81%) while, *B. orientalis* and *B. lateralis* comprised 8.11% and 11.7% respectively (Table 2). Diversity indices for this trimester were worked out for all species of cockroaches collected from different sites. Shannons index value (−0.51389) and Simpsons index of diversity was 0.6547 for this trimester. The dominant species of this period was *B. germanica* with a value of 0.196748 (Simpson index) and −0.1566 (Shannon index). During sampling the higher number of nymphs of late instar were found. Now they had gone through metamorphosis to change into adults and are ready for reproduction. In most species time for late instar to turn into females is longer than males.

In third trimester of study (October–December, 2013), *B. germanica* was most dominant species (44.62%) followed by *P. americana*, *B. orientalis* and *B. lateralis* respectively. Table 2 revealed Shannon index value (−0.51187) and Simpsons index of diversity (0.6521) for all species of cockroaches collected from different sites. Both species richness and abundance was significantly lower during colder months of study period.

The fourth trimester of study (January–March, 2014) Shannon index value was −0.52419 and Simpsons index of diversity was 0.6687*.* Most dominant species *B. germanica* (40.82%) was followed by *P. americana* (37.02%) and *B. orientalis* and *B. lateralis* comprise 7.17% and 14.97% respectively (Table 2). Overall species evenness and richness was found higher in second trimester of study (July–September, 2013).

Previous studies described *P. americana* and *B. orientalis* as outdoor species of cockroaches but they can intrude into indoor environment through severage pipes and crevices in harsh seasons. While *B. germanica* and *B. lateralis* always found in indoor environment (Rust and Reierson 2007, Jeffery et al. 2012). Residential areas and hospitals are mostly infected with *B. germanica*. *P. americana* approaches to outdoor environment through sewerage pipes and holes. As *B. orientalis* and *B. lateralis* are morphologically similar but their colony can be identified by male members. Houses and hospitals are highly infested with *P. americana* and *B. germanica* as compared to offices, shopping mall and institutes, whereas *B. orientalis* is commonly found in houses and institutes followed by shopping malls. Distribution of *B. lateralis* is most common in institutes, houses and offices with basements and gardens.

## Discussion

Environmental temperature plays an important role in determining the ability of an organism to survive in a given habitat. If an organism is able to survive in extreme temperature (during winter or summer) it will increases their likelihood to colonize that habitat. When an organism experience high or low temperature in their environment they produce certain proteins called “heat shock proteins (HSP)” in their cells which allows recovery on a cellular level. These HSP are found in many organisms from bacteria to mammals (Lutterschmidt and Hutchison 1997).

Organisms have the ability to increase or decrease their core temperature in response to environmental temperatures (Slabber et al. 2007). Certain insects, such as termites, have the ability to acclimate to their environmental temperature (Hu and Appel 2004). Since cockroaches are closely related to termites, they should have the tendency to acclimatize to their environment. Previous studies have shown a positive correlation between the temperature sensitivity of many animals including cockroaches and their environmental temperature (Tsuji and Mizumo 1973, Appel et al. 1983, Slabber et al. 2007).

In this study, cockroache’s species distribution was observed almost all around the year though environmental temperature may effect on distribution of some outdoor species. Cockroaches are highly adapted for diverse land environment especially dry harsh environment. Cockroaches were not more noticeable in cold months in third and fourth trimester but probably they have physical and behavioral adaptation which helps them to withstand in extreme low temperature on land. This study coincides with the findings of Snoddy and Appel (2008) who conducted a survey in Southern Alabama and Georgia to determine the extent *B. asahinai* had expanded its range Northward from Florida. They concluded that visual and bucket sample population began increasing in late May and reached their zenith in late August or early September.

The present study found indoor species of cockroaches including German and Turkestan cockroach were more prominent in bedrooms, kitchen, stores, hospital wards, office stores and rooms adjacent to canteen. Their high prevalence in these areas can be related to ideal shady, enriched food and cool environment which is more favorable to increase their population. Other outdoor species American and Oriental cockroaches are outdoor species and found near to sanitary pipes, washrooms, filthy habitat, adjacent gardens of houses, sewerage pipes and kitchen exit pipe where plenty food is available. Late nymphs and adults were more numerable in collection site where fewer females were captured. Environmental conditions like temperature and humidity also do affect the life cycle and developmental stages of cockroaches. Findings of our study are also in conformity to Lee et al. (2003) who found that houses were most commonly infested with *B. germanica* followed by apartments and villas. The infestation rate of the cockroaches was related to the residential types.

## Conclusion

Most of the urban areas in district Lahore especially human dwellings are infested with cockroach population. Their presence in human associated environment and species abundance pose them a critical threat for human health and indicate poor infrastructure of sanitary and waste disposal. The infestation rate of different species depends on availability of food sources, sanitary and climatic conditions. Cockroach infestation can be controlled with knowledge about their biology and behavior, attention to sanitation and effective use of commercial insecticides.
